# Integration of bioinformatics tools in candidate gene prioritization of co-regulated gene sets in *Saccharomyces cerevisiae*

**DOI:** 10.1186/1471-2105-12-S7-A18

**Published:** 2011-08-05

**Authors:** Vida Abedi, Mohammed Yeasin, Thomas R Sutter

**Affiliations:** 1 Department of Electrical and Computer Engineering; University of Memphis, Memphis, TN, 38152, USA; 2 Bioinformatics Program, College of Arts and Science; University of Memphis, Memphis, TN, 38152, USA; 3 Department of Biological Science, University of Memphis, Memphis, TN, 38152, USA

## 

The availability of massive amounts of heterogeneous and distributed biological data has prompted the development of a wide range of data analysis and data mining tools in the area of bioinformatics. However, due to the nature of the biological data, performing a specific analysis by combining such tools can be complicated and cumbersome. Yet, integration of number of tools can provide complementary information, and improve the efficiency of the data analysis to further our understanding and knowledge discovery. The development of an *integrated software platform* can considerably enhance the usability of such tools and benefits the research communities at large. Towards that goal, this study focuses on systematically integrating a number of tools for analyzing *Saccharomyces cerevisiae* data in order to improve candidate gene prioritization from microarray data using evidences from complementary sources.

Microarray data from a recent study by Ouyang *et. al.*[1] was used to evaluate the proposed framework. An array of free and open source bioinformatics tools were used to develop the *Saccharomyces Integrated Software Platform* (*SISP*). In particular, sources of information used in this analysis include literature data, Gene Ontology, physical and genetic interaction data as well as pathway information. SISP has the strength of combining prior knowledge with user-defined weighting of different sources of evidence. Access to the integrated tool will be facilitated by a user-friendly web interface with options including data query, import, export, analysis and visualization.

**Figure 1:**
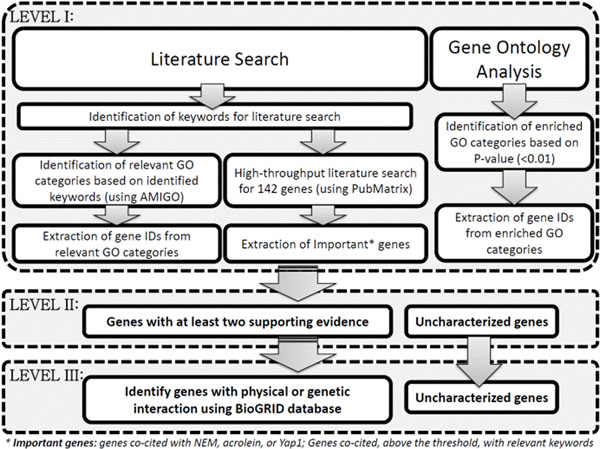
**Experimental design of the candidate gene prioritization process.** Information filtering is organized in three levels. At level 1, all the 142 genes are considered in the analysis. At the end of level 1, three sets of genes are obtained: 1) genes that are part of relevant GO categories, 2) genes for which there is significant amount of literature, and 3) genes that are part of enriched GO categories. All uncharacterized genes from the three lists are extracted and passed to the second level of prioritization. In addition, genes with at least two supporting evidences will also be forwarded to the second level of exploration. The filtered gene set from level 2 is used as input in level 3, where physical and genetic interaction among these genes are further explored. The resulting sets of genes will be the uncharacterized genes and the genes with at least two supporting evidences, which are then prioritized further if they are interrelated with at least one physical or genetic interaction.

The set of 142 genes from the microarray experiment was systematically reduced to sixteen genes (Figure 1); four out of the sixteen genes were highly ranked based on various sources of information. The sixteen genes were part of thirteen inter-related pathways, with eight genes playing major roles in those pathways. This integrated analysis enhanced extraction of essential information, and the identification of key inter-related pathways and genes. Integration of bioinformatics tools allows merging complementary sources of information which are critical to the identification of candidate genes for further experimental validation.
